# Macular Perfusion Impairment in Von Hippel-Lindau Disease Suggests a Generalized Retinal Vessel Alteration

**DOI:** 10.3390/jcm9082677

**Published:** 2020-08-18

**Authors:** Elisabetta Pilotto, Elisabetta Beatrice Nacci, Alfonso Massimiliano Ferrara, Gilda De Mojà, Stefania Zovato, Edoardo Midena

**Affiliations:** 1 Department of Ophthalmology, University of Padova, 35122 Padova, Italy; elisabetta.pilotto@unipd.it (E.P.); gildademo@gmail.com (G.D.M.); edoardo.midena@unipd.it (E.M.); 2 IRCCS—Fondazione G. B. Bietti, 00198 Rome, Italy; 3 Familial Tumor Unit, Veneto Institute of Oncology IOV-IRCCS, 35122 Padova, Italy; massimiliano.ferrara@iov.veneto.it (A.M.F.); stefania.zovato@iov.veneto.it (S.Z.)

**Keywords:** Von Hippel Lindau disease, retinal hemangioblastoma, OCT, OCT angiography, retinal vascular plexuses, macular flow

## Abstract

Background: To evaluate macular perfusion in patients with Von Hippel–Lindau (VHL) disease. Methods: VHL patients with or without peripheral retinal hemangioblastomas (RHs) were consecutively enrolled. A group of healthy subjects served as controls. Macular perfusion was analyzed by means of OCT angiography (OCTA) in the superficial vascular plexus (SVP), and in the intermediate (ICP) and deep retinal capillary (DCP) plexuses. The following OCTA parameters were measured: Vessel Area Density (VAD), Vessel Length Fraction (VLF), Vessel Diameter Index (VDI) and Fractal Dimension (FD). Results: Sixty-three VHL patients (113 eyes) and 28 healthy controls (56 eyes) were enrolled. All OCTA quantitative parameters were reduced in VHL patients vs. controls, reaching statistical significance for VAD of the SVP (0.348 ± 0.07 vs. 0.369 ± 0.06, *p* = 0.0368) and VDI of all plexuses (*p* < 0.03 for all). No significant differences were detected between eyes without or with peripheral RHs. Conclusions: Macular perfusion is reduced in VHL patients demonstrating retinal vessel changes that are independent of the presence of peripheral RHs. VHL gene mutations disrupt the hypoxia-induced (HIF)/vascular endothelium growth factors (VEGF) pathway and the Notch signaling, both essential for the normal retinal vasculogenesis and angiogenesis. Therefore, an anomalous generalized retinal vascular development may be hypothesized in VHL disease.

## 1. Introduction

Von Hippel–Lindau (VHL) disease (OMIM 193300) is an autosomal dominant heritable cancer syndrome with an incidence of 1 in 36,000 live births per year, caused by the oncosuppressor VHL gene mutation. In VHL disease, benign and malignant tumors and/or cysts develop throughout the central nervous system (CNS) and visceral organs (clear cell renal carcinoma, pheochromocytomas, neuroendocrine tumors and cysts of the pancreas, endolymphatic sac tumors, papillary cystadenomas of the epididymis and broad ligament) [1]. The mean age of onset is in the third decade of life and by the age of 65 years 90% of patients become symptomatic. Eugen von Hippel, a German ophthalmologist, first described retinal lesions (“angiomatosis retinae”) in 1904 while Arvid Lindau, a Swedish pathologist, recognized the association between retinal (RHs) and cerebellar hemangioblastomas (CHs) in 1927 [2,3]. RHs are well-circumscribed reddish-orange vascular lesions characterized by a tortuous feeding artery and draining vein that develop in the peripheral retina or in the iuxtapapillary area [1]. Small RHs are asymptomatic, while larger lesions can cause macular edema, exudative or tractional retinal detachment and vitreous bleeding [4]. By means of widefield fluorescein angiography, RH-associated peripheral retinal nonperfusion has been recently described in eyes with peripheral RH, suggesting a vascular steal phenomenon from the surrounding normal retina [5]. With the introduction in clinical practice of optical coherence tomography angiography (OCTA), it has now become possible to explore in vivo, without any dye, the central and midperipheral retinal blood flow. Moreover, OCTA allows separate analysis of three single retinal vascular plexuses: the superficial vascular plexus (SVP), and two deeper capillary plexuses: the intermediate (ICP) and the deep capillary plexus (DCP), invisible to fluorescein angiography [6,7]. The aim of this study was to investigate macular perfusion, using OCTA, in VHL patients with or without peripheral RHs.

## 2. Experimental Section

### 2.1. Participants

In this cross-sectional study, all patients with VHL disease genetically confirmed of the Familial Tumor Unit, Veneto Institute of Oncology (IOV-IRCCS) undergoing scheduled eye examination, were consecutively recruited. All patients underwent complete ophthalmological evaluation including: best-corrected visual acuity (BCVA), using standard Early Treatment Diabetic Retinopathy Study (ETDRS) charts; slit-lamp examination of the anterior segment; intraocular pression measurement; indirect ophthalmoscopy; and 90-diopter-lens biomicroscopy. Exclusion criteria were: RHs or laser scars from previous treatments or retinal pigment epithelium changes involving the posterior pole; epiretinal macular membrane; history of inflammatory ocular diseases; concomitant presence of other retinal vascular diseases; congenital or acquired anterior segment disorders; glaucoma; and refractive errors ≥ 6 diopters. The same examinations were performed on an age-matched healthy group for comparison. The approval from the Ethics Committee for Clinical Practice of the Azienda Ospedaliera di Padova for the study was obtained (Prot. 34971/AOP/2018). Informed consent was obtained from each subject and data collection followed the tenets of the Declaration of Helsinki.

### 2.2. OCT and OCT Angiography

Optical coherence tomography (OCT) and OCTA were performed using Spectralis HRA + OCTA (Heidelberg Engineering, Heidelberg, Germany). All scans were acquired after pupil dilatation, obtained with 1% tropicamide solution, in a dark room, late in the morning, and the in-built eye-tracker always activated. The scan protocol included a single horizontal scan (180° line scan, 9-mm length, automated real time (ART) set at 100 frames), a macular map of 20° × 20° (5.8 × 5.8 mm; 97 B-scans separated by 60 micron) and an OCTA scan pattern of 10° × 10° (3.0 × 3.0 mm; 512 B-scans separated by 6 micron) all centered onto the fovea. The inbuilt software automatically generated the enface OCTA images of the full retina (extending from the inner limiting membrane to the Bruch membrane) and of the SVP, ICP and DCP, as automatically segmented by the device, to avoid segmentation manual errors. For high quality OCTA images, a signal strength (SS) more than 30 in “Q score” (on a scale of 0 to 40 for Spectralis, Heidelbeerg, Germany) was required [8,9]. An eye tracking system was used to guarantee correct foveal centration of the scans. A skilled technician performed all scans and checked each image after acquisition to detect any motion artifacts or segmentation errors and eventually repeated examination.

### 2.3. OCTA en Face Image Analysis

**Figure 1:**
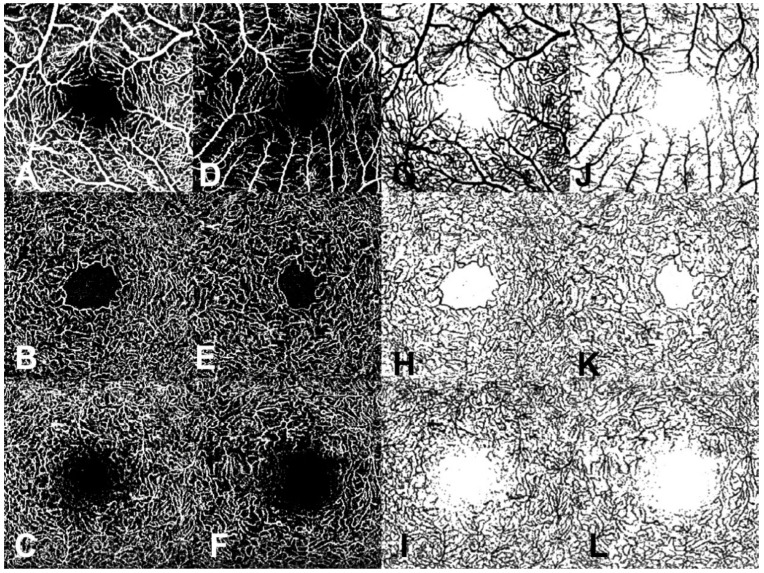
En-face OCT angiography (OCTA) and corresponding binary images of an eye of a Von Hippel–Lindau (VHL) patient (**D**–**F**,**J**–**L**) and of a healthy control (**A**–**C**,**G**–**I**) of the superficial vascular plexus (SVP, **A**,**D**), of the intermediate capillary plexus (ICP, **B**,**E**) and of the deep capillary plexus (DCP, **C**,**F**). In the VHL eye a reduction of the vessel density is detectable, mainly at the SVP level.

Quantitative analysis of the OCTA en face images was performed using an open-source available ImageJ software (National Institutes of Health, Bethesda, MD, USA). The foveal avascular zone (FAZ) was measured in the en face OCTA image of the full retina as previously suggested [7]. The following quantitative parameters were analyzed in each vascular plexus (SVP, ICP and DCP): Vessel Area Density (VAD), Vessel Length Fraction (VLF), and Vessel Diameter Index (VDI) [10]. Briefly, OCTA en face images were automatically converted into a binary image (Figure 1). VAD was obtained dividing the number of black pixels in the binary image by the total number of image pixels. A skeletonized image was then automatically elaborated from the binarized one by iteratively deleting the outer boundary of the binarized image until a single pixel remains for each vessel segment. VLF was obtained dividing the number of vessel pixels in the skeletonized image by the total number of image pixels. VDI was obtained by processing both the binary and the skeletonized images to calculate the average vessel caliber. Moreover, Fractal Dimension (FD), representing complexity of images, was calculated both over the skeletonized images (FDsk) using a box counting technique and over binary images (FDbin) [10,11].

### 2.4. Statistical Evaluation

All variables were summarized according to the usual methods of descriptive statistics: mean and standard deviation for quantitative variables, and absolute and relative (percentage) frequencies for qualitative variables. Chi-square was used to compare gender distribution and t-Student test for age. Three groups of comparison were defined: (1) VHL patients vs. controls; (2) VHL eyes with RH vs. VHL eyes without RH; (3) eyes with RH vs. fellow eyes without RH in patients with monocular lesions. All the models used in these statistical analyses accounted for replication of measures in both eyes (PROC MIXED of SAS 9.4 statistical software). CRT and OCTA parameters were compared between groups using a mixed effects two-way ANOVA model, with repeated measures (both eyes) and adjusted for age. Bonferroni post-hoc test was used to compare adjusted mean values between groups within the same plexus. Correlation between OCTA parameters and age and CRT was analyzed by means of a generalized linear regression model. Estimation of regression coefficients and their statistical significance was chosen as marker of correlation. Control subjects were recruited on a voluntary basis. The total sample size of enrolled subjects was calculated in order to recognize differences of at least 18% of the reference values as statistically significant, using a 2-sided t test with type I error alpha = 0.05, and a power of 0.80. Moreover, an enrollment ratio of 2.25 patients per 1 control was considered adequate for comparison within subgroups. In addition, since both eyes of patients and controls contributed to the study, the sample size was adjusted applying the factor 1/(1-rho), where rho = 0.60 is the estimated correlation between eye measures.

Statistical significance was set at *p* < 0.05. All the analyses were performed by SAS^®^ 9.4 statistical software (SAS Institute, Cary, NC, USA).

## 3. Results

Sixty-three VHL patients and 28 healthy controls were consecutively enrolled from December 2018 to January 2020. All subjects were Caucasian. Patients and controls did not differ for mean age and sex distribution. BCVA was significantly lower in VHL patients than in controls (84.4 ± 8.4 letters vs. 87.8 letters ± 3.7 letters, *p* = 0.0003) (Table 1).

In 13 patients only one eye was included; the fellow eye was excluded because of: RH at the posterior pole (5 cases), band keratopathy (2 cases), previous total retinal detachment related to RH (1 case), epiretinal macular membrane (3 cases), myelinated nerve fibers (1 case), and retinal pigment epithelium changes involving the macula (1 case). Therefore, 113 eyes of 63 VHL patients (71 eyes without RHs (62.8%) and 42 (37.1%) with peripheral RHs) and 56 eyes of 28 healthy subjects were included in the analysis.

Sixteen patients (25.4%) had only one eye affected by peripheral RHs. Mean SS of the OCTA images was 37.53 ± 2.76 “Q score” in VHL patients and 38.18 ± 3.34 “Q score” in controls. CRT and FAZ were not significantly different between VHL patients and controls (273.9 ±17.5 µm vs. 272.7 ± 18.0 µm, *p* = 0.8194 and 0.30 ± 0.11 mm^2^ vs. 0.32 ± 0.09 mm^2^, *p* = 0.3637; respectively). All the other OCTA parameters were reduced in VHL patients versus controls, reaching statistical significance for the VAD of the SVP (0.348 ± 0.07 vs. 0.369 ± 0.06, *p* = 0.0368), and for the VDI of all remaining plexuses (*p* < 0.02 for all) (Table 2 and Figure 1).

CRT and OCTA parameters were not significantly different between eyes without (71 eyes) or with RHs (42 eyes) (Table 3).

In patients with monocular lesions, there were no differences between the eyes with RHs and the fellow eyes without RHs (Table 4).

## 4. Discussion

In this study, macular perfusion impairment was detected in patients with VHL disease and no RHs at the posterior pole using OCTA. The main vascular changes were detectable at the SVP level, where the vascular area density (VAD) and the vessel diameter index (VDI) were significantly reduced, depicting a vascular bed characterized by retinal vessels lower in number and thinner in diameter compared to healthy controls. Vascular changes were independent of the presence of peripheral RHs. Recently, Pulido et al, using widefield fluorescein angiography, reported peripheral retinal nonperfusion in eyes with peripheral RH associated with VHL disease [12]. The same authors confirmed this finding in a multicenter larger case series of eyes with RH, related or not to VHL disease [5]. They hypothesized a vascular steal phenomenon, with larger tumors stealing more blood from the surrounding normal retina [5]. In the present study, only VHL patients, with no vascular or degenerative changes at the posterior pole, were consecutively studied. Retinal perfusion was investigated in the macular area, far from existing RHs and macular flow changes were independent of their presence. Therefore, a different phenomenon, rather than simple steal, may be hypothesized, at least in VHL patients, to explain vascular changes and a reduction of macular blood flow may be hypothesized. Moreover, in the previously reported angiographic study, vascular nonperfusion—both anterior and posterior to the tumor—was present in 63% of the VHL patients, and in none without VHL [5]. Therefore, a generalized retinal vessel defect may be present in VHL patients as an effect of the VHL gene mutations, which affect both retinal vasculogenesis and angiogenesis. In a retina-specific conditional knockout VHL animal model, poorly-formed retinal vessels, with excessive vessel regression, have been described [13]. In the present study, vascular area density (VAD) was reduced, confirming in humans the decrease of the retinal vascular bed. In animal models of specific VHL gene mutation or loss, aberrant increase in arterial maturation, smaller diameter arterioles and arterial branches directly connected to venules have also been reported [13,14]. These findings may explain the more relevant vascular changes detected at the SVP, mainly composed of horizontal arterioles and venules connected by transverse capillaries, than at the deeper capillary plexuses, composed of polygonal lobules of capillaries [7,15,16]. The FAZ, surrounded by short capillaries directly interconnecting arteries and veins [17], seems not to be affected. The vessel diameter (VDI at OCTA) reduction in VHL patients versus controls was 2.7% for both SVP and DCP and 2.8% for ICP. These differences seem to be small. However, VDI is linearly related to the internal vessel diameter [11]. As a consequence, the calculated vessel cross sectional area differences are 5.2% for SVP, 10% for ICP, and 3.8% for DCP. These differences, even if not directly comparable, are relevant compared to other retinal conditions affecting vessels diameter and blood flow, as systemic hypertension or central nervous system vascular neurodegenerative diseases [18,19]. Therefore, VHL disease needs to be better investigated in a scenario of complex disorders involving microvasculature.

OCT angiography is a new retinal imaging modality that has allowed detection of microvascular changes and reduced macular vessel densities in different vascular and neurodegenerative disorders [20,21]. In VHL disease, OCTA had been previously used to identify tiny posterior RHs, to better define intrinsic vasculature and feeder vessels in iuxtapapillary tumors, and to detect the activity of previously treated RHs or choroidal neovascularization secondary to laser scars [22,23].

We used OCTA to investigate macular perfusion in a large VHL population, detecting a reduction in macular blood flow. Our findings differ from those recently found, where an increase in macular vessel density in VHL patients was detected in both SCP and DCP [24]. However, our results cannot be easily compared when different OCTA commercial software is used and the ICP is partially incorporated into the other plexuses. Moreover, in the present study we excluded VHL eyes with any lesion and/or changes at the posterior pole, including the epiretinal membrane, that seems to increase macular flow [25].

One of the main limits of this study is the lack of peripheral retinal perfusion data to be correlated to macular flow. This is due to the intrinsic limitation of OCTA, which is currently inadequate to evaluate peripheral retina perfusion. In this study, all VHL patients referred for a routine ophthalmological examination were consecutively recruited. Therefore, fluorescein angiography, an invasive test, was performed only in some cases (11 patients, 17.4%), and the small sample size did not allow performance of any separate analysis. The identification of RHs was clinically performed. Therefore, the presence of tiny clinically undetectable RHs, visible just with fluorescein angiography, cannot be excluded. Another limitation is that genotype-phenotypes correlation was not performed [4,5,6,7,8,9,10,11,12,13,14,15,16,17,18,19,20,21,22,23,24,25,26]. Therefore, we cannot exclude that the genotype of VHL germline mutation may differently influence retinal vascular development and perfusion. A further analysis of genotype-OCTA parameters correlations is ongoing.

In conclusion, this study describes an abnormal retinal vascular pattern of the macula, characterized by reduction in macular perfusion in VHL patients, independently of peripheral RHs presence. This allows to hypothesize an anomalous vascular development induced by VHL gene mutations as VHL protein is a key factor in both ocular vasculogenesis and angiogenesis.

## Figures and Tables

**Table 1:** Demographic characteristics of enrolled von Hippel-Lindau patients and healthy controls.

	VHL Patients	Controls	*p* Value
**Subjects, (number)**	63	28	
**Mean age (years: mean, SD)**	40.5, 14.4	40.0, 11.8	0.8824
**Female:Male (number, %)**	36:27, 57.1:42.9	17:11, 60.7:39.3	0.7498
**BCVA (letters: mean, SD)**	84.4, 8.4	87.8, 3.7	**0.0003**
**Peripheral RH (number, %)**			
**Present**	36, 57	-	-
**Absent**	27, 43	-	-

VHL: Von Hippel–Lindau disease; BCVA: best corrected visual acuity; RH: retinal hemangioblastoma; *p* value: statistically significant values in bold.

**Table 2:** OCT and OCT angiography parameters in von Hippel-Lindau patients and healthy controls.

Subjects	VHL Patients	Controls	*p* Value
(number, %)	(63, 69.2)	(28, 30.7)	
**CRT (mm: mean ± SD)**	273.9 ±17.5	272.7 ±18.0	0.8194
**FAZ (mm^2^: mean ± SD)**	0.30 ± 0.11	0.32 ± 0.09	0.3637
**VAD (mean ± SD)**			
**SVP**	0.3476 ± 0.07	0.3685 ± 0.06	**0.0368**
**ICP**	0.1517 ± 0.03	0.1653 ± 0.03	0.1536
**DCP**	0.1905 ± 0.04	0.2067 ± 0.05	0.0942
**VLF (mean ± SD)**			
**SVP**	0.0789 ± 0.01	0.0815 ± 0.01	0.2476
**ICP**	0.0497 ± 0.01	0.0528 ± 0.01	0.1824
**DCP**	0.0589 ± 0.01	0.0622 ± 0.01	0.1462
**VDI (mean ± SD)**			
**SVP**	4.4038 ± 0.23	4.5237 ± 0.24	**0.0012**
**ICP**	3.0218 ± 0.16	3.1087 ± 0.14	**0.0159**
**DCP**	3.2056 ± 0.17	3.2931 ± 0.16	**0.0153**
**FDbin (mean ± SD)**			
**SVP**	1.7470 ± 0.07	1.7655 ± 0.05	0.1659
**ICP**	1.5546 ± 0.08	1.5761 ± 0.07	0.1160
**DCP**	1.6190 ± 0.07	1.6367 ± 0.08	0.1819
**FDsk (mean ± SD)**			
**SVP**	1.5052 ± 0.07	1.5174 ± 0.05	0.2985
**ICP**	1.3795 ± 0.07	1.3957 ± 0.07	0.1883
**DCP**	1.4353 ± 0.06	1.4481 ± 0.07	0.2781

VHL: Von Hippel-Lindau Disease; RH: retinal hemangioblastoma; CRT: central retinal thickness; FAZ: foveal avascular zone; VAD = vascular area density; VLF = vessel length fraction; VDI = vessel diameter index; FDbin: fractal dimension of binary image; FDsk: fractal dimension of skeletonized images; SVP = superficial vascular plexus; ICP = intermediate capillary plexus; DCP = deep capillary plexus; *p* value: statistically significant values in bold.

**Table 3:** Best corrected visual acuity, OCT and OCT angiography parameters in VHL eyes with or without peripheral retinal hemangioblastomas (RHs).

VHL Eyes	With RHs	w/Out RHs	*p* Value
**(number, %)**	(42, 37.1)	(71, 62.8)	
**BCVA (letters: mean ± SD)**	85.29 ± 2.08	85.01 ± 2.60	0.1809
**CRT (m: mean ± SD)**	273.8 ± 18.5	273.9 ± 17.0	0.3011
**FAZ (mm^2^: mean ± SD)**	0.31 ± 0.12	0.29 ± 0.10	0.1302
**VAD (mean ± SD)**			
**SVP**	0.3486 ± 0.06	0.3470 ± 0.08	0.9510
**ICP**	0.1503 ± 0.03	0.1526 ± 0.03	0.6872
**DCP**	0.1838 ± 0.04	0.1945 ± 0.04	0.1661
**VLF (mean ± SD)**			
**SVP**	0.0789 ± 0.01	0.0789 ± 0.01	0.9245
**ICP**	0.0492 ± 0.01	0.0501 ± 0.01	0.7261
**DCP**	0.0570 ± 0.01	0.0600 ± 0.01	0.1626
**VDI (mean ± SD)**			
**SVP**	4.4188 ± 0.27	4.3948 ± 0.21	0.7534
**ICP**	3.0259 ± 0.17	3.0194 ± 0.16	0.8362
**DCP**	3.1962 ± 0.17	3.2113 ± 0.16	0.3958
**FDbin (mean ± SD)**			
**SVP**	1.7520 ± 0.05	1.7440 ± 0.08	0.7727
**ICP**	1.5527 ± 0.07	1.5558 ± 0.08	0.4647
**DCP**	1.6109 ± 0.06	1.6239 ± 0.08	0.1018
**FDsk (mean ± SD)**			
**SVP**	1.5096 ± 0.05	1.5026 ± 0.08	0.7254
**ICP**	1.3774 ± 0.06	1.3807 ± 0.07	0.5236
**DCP**	1.4277 ± 0.05	1.4399 ± 0.07	0.1346

VHL: Von Hippel Lindau Disease; RH: retinal hemangioblastoma; BCVA: best corrected visual acuity; FAZ: foveal avascular zone; CRT: central retinal thickness; VAD = vascular area density; VLF = vessel length fraction; VDI = vessel density index, FDbin: fractal dimension of binary image; FDsk: fractal dimension of skeletonized images; SVP = superficial vascular plexus; ICP = intermediate capillary plexus; DCP = deep capillary plexus; *p* value: statistically significant values in bold.

**Table 4:** OCT and OCTA parameters in VHL patients with monocular lesions (eyes with RHs versus fellow eyes with no RHs).

VHL Patients	Eyes with RHs	Fellow Eyes	*p* Value
**(16 cases)**			
**BCVA (letters: mean ± SD)**	85.31 ± 1.74	85.75 ± 2.70	0.2760
**CRT (m: mean ± SD)**	275.1 ± 17.7	277.0 ± 19.4	0.3669
**FAZ (mm^2^: mean ± SD)**	0.31 ± 0.14	0.32 ± 0.13	0.3313
**VAD (mean ± SD)**			
**SVP**	0.3193 ± 0.07	0.3378 ± 0.06	0.0770
**ICP**	0.1375 ± 0.04	0.1423 ± 0.03	0.6418
**DCP**	0.1858 ± 0.06	0.1819 ± 0.05	0.7066
**VLF (mean ± SD)**			
**SVP**	0.0726 ± 0.01	0.0768 ± 0.01	0.1212
**ICP**	0.0456 ± 0.01	0.0469 ± 0.01	0.6249
**DCP**	0.0577 ± 0.01	0.0566 ± 0.01	0.6756
**VDI (mean ± SD)**			
**SVP**	4.4038 ± 0.24	4.4056 ± 0.22	0.9734
**ICP**	2.9578 ± 0.21	2.9974 ± 0.19	0.4550
**DCP**	3.1718 ± 0.21	3.1811 ± 0.17	0.8612
**FDbin (mean ± SD)**			
**SVP**	1.7207 ± 0.07	1.7420 ± 0.05	0.1678
**ICP**	1.5170 ± 0.11	1.5340 ± 0.08	0.2715
**DCP**	1.6082 ± 0.08	1.6037 ± 0.08	0.7724
**FDsk (mean ± SD)**			
**SVP**	1.4787 ± 0.07	1.4993 ± 0.05	0.1626
**ICP**	1.3467 ± 0.09	1.3599 ± 0.07	0.3695
**DCP**	1.4266 ± 0.07	1.4210 ± 0.07	0.7002

VHL: Von Hippel Lindau Disease; RH: retinal hemangioblastoma; BCVA: best corrected visual acuity; CRT: central retinal thickness; FAZ: foveal avascular zone; VAD = vascular area density; VLF = vessel length fraction; VDI = vessel diameter index; FDbin: fractal dimension of binary image; FDsk: fractal dimension of skeletonized images; SVP = superficial vascular plexus; ICP = intermediate capillary plexus; DCP = deep capillary plexus; *p* value: statistically significant values in bold.
